# Technological and Genomic Analysis of Roles of the Cell-Envelope Protease PrtS in Yoghurt Starter Development

**DOI:** 10.3390/ijms19041068

**Published:** 2018-04-03

**Authors:** Hui Tian, Bailiang Li, Smith Etareri Evivie, Shuvan Kumar Sarker, Sathi Chowdhury, Jingjing Lu, Xiuyun Ding, Guicheng Huo

**Affiliations:** 1 Key Laboratory of Dairy Science, Ministry of Education, Food College, Northeast Agricultural University, Harbin 150030, China; ytntianhui@yili.com (H.T.); bailiangli@neau.edu.cn (B.L.); smith.evivie@uniben.edu (S.E.E.); Shuvan.sarker@gmail.com (S.K.S.); sathichowdhury85@gmail.com (S.C.); lujj0324@gmail.com (J.L.); aidingxiuyun@outlook.com (X.D.); 2 Food Science and Nutrition Unit, Department of Animal Science, Faculty of Agriculture, University of Benin, Benin City PMB 1154, Nigeria; 3 Guangzhou Genedenovo Biotechnology Co. Ltd., Guangzhou 510000, China

**Keywords:** *Streptococcus thermophilus*, *Lb*. *bulgaricus*, proteolysis, technological, genomics

## Abstract

The cell-envelope protease PrtS was proved to be efficient in optimal bacterial growth and fast acidification in pure culture, while its positive effect on the performance of mixed-cultures in milk fermentation was not defined. The aim was to analyze effects of the PrtS on the symbiosis between strains during yoghurt production and cold storage. Two *Streptococcus thermophilus* strains, KLDS3.1012 and KLDS SM, and two different proteolytic strains of *Lactobacillus delbrueckii* subsp. *Bulgaricus*, L7 and L12, were used. Technological properties (viability, acid production, and proteolysis) were determined. Comparative genomics was used to analyze the proteolytic system (cell-envelope protease, transport system, intracellular peptidase) of *Streptococcus thermophilus* strains. *S*. *thermophilus* KLDS SM possesses an intact gene encoding PrtS (A9497_00420), which was not found in the genome of *S. thermophilus* KLDS3.1012. This gene is the main difference in the proteolytic system between the two genomes. PrtS endowed KLDS SM high levels of viability during fermentation and cold storage. When combined with a weaker lactobacillus strain during fermentation, the acceleration of acid production of mixed-culture by KLDS SM would start at an earlier time. KLDS SM increased the post-acidification of yoghurts during cold storage, but the pH was steadily maintained during 14–28 days. Results suggest that strains of *Streptococcus thermophilus* with strong proteolytic ability could be used in a wide range of dairy production. The present study provided data for yoghurt starter development from the point of view of proteolysis.

## 1. Introduction

Yoghurt is manufactured using a co-culture of *Streptococcus thermophilus* (*S. thermophilus*) and *Lactobacillus delbrueckii* subsp. *bulgaricus* (*Lb. bulgaricus*) at 42 °C up to pH 4.5 or below, followed by the storage at 4–10 °C [1]. Both species are Gram-positive, anaerobic homofermentative, and thermophilic (capable of growing at 40–45 °C) lactic acid bacteria [2]. The positive interaction between *S. thermophilus* and *Lb. bulgaricus* in yoghurt production called “proto-cooperation”, leads to a stimulation of their growth, acid production and confers desirable rheological properties [3].

Milk contains lactose (45–50 g·L^−1^) and caseins (~80% of milk proteins). However, its concentration of free amino acids and short peptides is too low to support the rapid growth of yoghurt strains [4,5]. An efficient proteolytic system becomes a viable alternative because it can contribute to the rapid growth and fast acidifying ability of strains, good texture, flavor and modification of other milk nutrients [6,7,8,9]. It, thus, follows that the selection of strains possessing efficient proteolytic systems may play an important role in yoghurt starter optimization.

PrtS and PrtB are cell-envelope proteases (CEP) found in strains of *S. thermophilus* and *Lb. bulgaricus*, respectively, and are capable of initiating the breakdown of caseins into oligopeptides. Both of them are serine proteases, which belong to the subtilisin-like serine protease family known as the subtilase family [7,10]. Large variances of proteolytic activities have been reported among strains of *S. thermophilus* and *Lb. bulgaricus* [11]. *Lb. bulgaricus* generally possesses strong proteolytic activity, while only a few strains of *S. thermophilus* having PrtS displayed a high level of proteolytic activity. In addition, PrtS endowed strains are characterized with fast growth ability and rapid acid production in pure culture in milk [5,12,13]. A natural transformation method was used to obtain rapid acidifying ability in *S. thermophilus* by transforming of the protease gene *prtS* [14]. Roles of different proteolytic strains in associative fermentations have also been studied previously and these were shown to be strain-specific among other factors [13]. Protease of *Lb. bulgaricus* (PrtB) was involved in the optimal growth of *S. thermophilus*, and affected the association on milk acidification, whereas PrtS was not essential in the mixed culture [15]. However, the proteolytic phenotypes highly depend on strains selected in the association, revealing that more studies are required especially as new strains possessing different proteolytic abilities are being explored. Furthermore, studies reporting the post-acidification of yoghurt prepared simultaneously by different proteolytic strains are scarce, thus, more research is needed for understanding the relationship between post-acidification and proteases of yoghurt strains.

The aim of this research was to investigate the effect of proteolytic systems on yoghurt starter development, using two strains of *S. thermophilus* KLDS SM and KLDS3.1012 (*prtS*+ and *prtS*−) and two *Lb. bulgaricus* strains, L7 and L12 (proteolysis-strong and weak). Technological properties of the four bacterial associations during 12-h fermentation and cold storage were analyzed. Genomic comparison of the proteolytic system between two *S. thermophilus* strains was carried out to find more differences at the genomic level.

## 2. Results and Discussion

### 2.1. Technological Properties of S. thermophilus and Lb. bulgaricus

**Figure 1:**
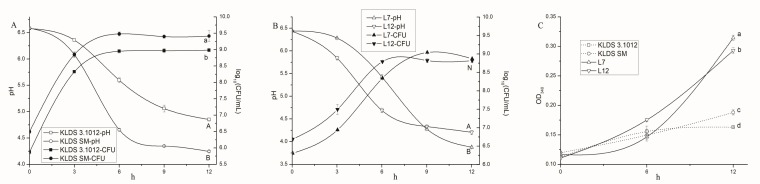
Technological properties of *S. thermophilus* and *Lb. bulgaricus* strains in pure culture: (**A**) pH value of milk and viability of *S. thermophilus* strains KLDS3.1012 and KLDS SM during 12-h fermentation; (**B**) pH value of milk and viability of *Lb. bulgaricus* strains L7 and L12 during 12-h fermentation; and (**C**) Proteolysis (Optical density, OD340 nm) of four strains of *S. thermophilus* and *Lb. bulgaricus* at 0, 6, and 12 h during milk fermentation in pure culture. N: no significant difference for live counts.

The variability in acid production, growth, and proteolytic ability between strains of *S. thermophilus* as well as between strains of *Lb. bulgaricus* were reported previously [5,16,17]. Acid production and growth of *S. thermophilus* (Figure 1A) and *Lb. bulgaricus* (Figure 1B) were monitored by the pH and viable counts at 0, 3, 6, 9, and 12 h respectively. The proteolytic activity (OD340) of strains in milk at 0, 6, 12 h was obtained (Figure 1C).

#### 2.1.1. Acid Production and Growth

A rapid pH decline started at ~3 h in the four samples of *S. thermophilus* and *Lb. bulgaricus*, but ended at different times. KLDS SM displayed faster acid production and growth rate than KLDS3.1012. The acid production rate of L12 was faster than L7 before 9 h but decreased subsequently, while L7 kept acid production until pH 3.88. Similarly, the growth of L12 was faster than L7 before 6 h, but no significant difference (*p* > 0.05) was found at 12 h between the two strains.

It is known that *S. thermophilus* usually produces less amounts of acid in milk than *Lb. bulgaricus* [5]. Due to less tolerance to low pH, *S. thermophilus* does not grow well in the culture below pH 4.4 [18]. This observation is in consonance with our study. The growth of yoghurt strains largely depends on adequate free amino acids and peptides supplied by their proteolytic systems, which are different between strains [5].

#### 2.1.2. Proteolytic Activity

The proteolytic activities of *Lb. bulgaricus* were strongly higher than *S. thermophilus* during 6–12 h. The absorbance value of KLDS SM was higher than KLDS3.1012 at 12 h. The previous reports suggested that *S. thermophilus* was typically defined as low proteolytic species, and consumed the amino acids and small peptides initially present in whey to support its optimal growth [17]. In addition to the favorable initial pH of milk, *S. thermophilus* is a better competitor for utilization of limiting nutrients in milk than *Lb. bulgaricus*, probably leading to the rapid growth of *S. thermophilus* in pure culture and its dominant population in mixed culture with *Lb. bulgaricus* [5,13,19]. A membrane protease gene (part region of *prtS* gene) was found in all four fast-acidifying *S. thermophilus* strains, which also displayed superior growth on milk agar [20].

### 2.2. General Features of Genomes of S. thermophilus

**Figure 2:**
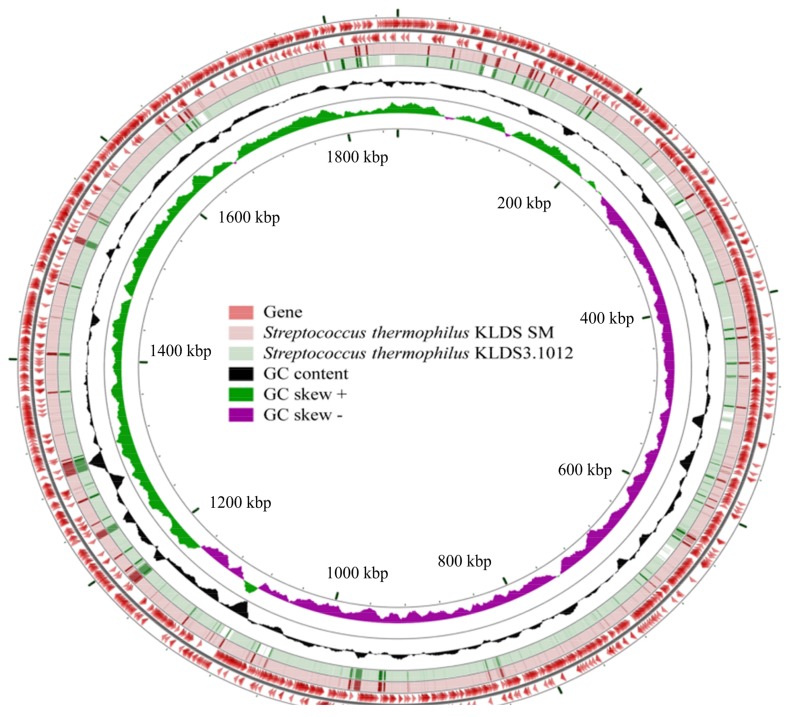
Circular genome map of *S. thermophilus* KLDS SM and KLDS3.1012. From outside to inside: Circles 1 and 2 showed the locations of genes, including protein coding genes CDS, rRNA, tRNA and other genes on positive and negative chain; Circles 3–4 showed the comparisons of *S. thermophilus* KLDS SM and *S. thermophilus* KLDS3.1012 through blastn; Circles 5 and 6 showed GC content and GC skew.

In order to mine the differences of molecular basis for proteolytic activity and acid producing properties of *S. thermophilus* KLDS SM and *S. thermophilus* KLDS3.1012 from genomic perspective, the genomes of these two strains were sequenced and investigated in silico. A comparison of general features of these two strains is shown in Figure 2, the genome sequences of *S. thermophilus* KLDS SM and *S. thermophilus* KLDS3.1012 contain one scaffold (1,856,787 bp length with mean G + C content of 39.08%) and ten scaffolds (1,853,856 bp length with mean G + C content of 39.20%), respectively. No plasmids were found in the genomes of these two strains. *S. thermophilus* KLDS3.1012 possessed more numbers of 1992 and 213 of genes and pseudogenes than the numbers of *S. thermophilus* KLDS SM (1950 genes and 129 pseudogenes), respectively. However, low numbers of 1705 protein-coding genes (CDS) and 74 RNA genes were identified in the genome of *S. thermophilus* KLDS3.1012, compared with *S. thermophilus* KLDS SM (1732 CDS and 89 RNA genes).

**Figure 3:**
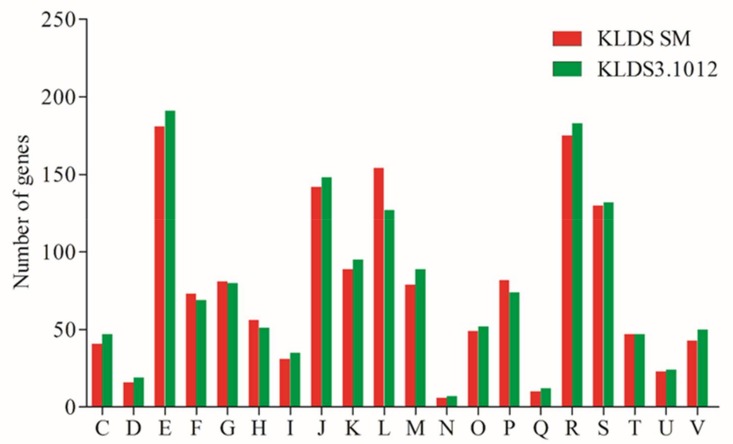
COG functional classification of protein-coding gene in *S. thermophilus* KLDS SM and *S. thermophilus* KDLS3.1012 genome. C: Energy production and conversion; D: Cell cycle control, cell division, chromosome partitioning; E: Amino acid transport and metabolism; F: Nucleotide transport and metabolism; G: Carbohydrate transport and metabolism; H: Coenzyme transport and metabolism; I: Lipid transport and metabolism; J: Translation, ribosomal structure and biogenesis; K: Transcription; L: Replication, recombination and repair; M: Cell wall/membrane/envelope biogenesis; N: Cell motility; O: Posttranslational modification, protein turnover, chaperones; P: Inorganic ion transport and metabolism; Q: Secondary metabolites biosynthesis, transport and catabolism; R: General function prediction only; S: Function unknown; T: Signal transduction mechanisms; U: Intracellular trafficking, secretion, and vesicular transport; V: Defense mechanisms.

Phenotypic differences between *S. thermophilus* KLDS SM and *S. thermophilus* KLDS3.1012 may be predicted by distinction in the number of genes ascribed to clusters of orthologous groups (COG) functional annotation. The result of gene abundance of all subcategories of these two strains is presented in Figure 3. A total of 1508 CDS (87.07%) and 1532 CDS (89.85%) were assigned to different COG categories from class C to class V, respectively. Furthermore, the three highest variable categories were assigned into subcategories: (L) “Replication, recombination and repair”, (E) “Amino acid transport and metabolism”, and (M) “Cell wall/membrane/envelope biogenesis”. No major differences were found in other functional groups, this indicates that the above three variable groups may be closely related to different properties of *S. thermophilus*. As we focused on proteolytic and acidifying abilities of *S. thermophilus*, class E was analyzed in silico in detail.

### 2.3. Proteolytic System and Amino Acid Metabolism of S. thermophilus

The proteolytic system of *S. thermophilus* mainly consists of (i) an extracellular cell-envelope protease capable of casein breakdown; (ii) a set of transport systems for import of amino acids and oligopeptides; and (iii) a pool of various intracellular peptidases involved in the hydrolysis for casein-derived peptides [21,22]. Genetic elements of the proteolytic system in the genomes of *S. thermophilus* KLDS SM and *S. thermophilus* KLDS3.1012 were determined from these three aspects by bioinformatics analysis.

As shown in Table 1, the genomes of these two strains unveiled the presence of a variety of proteases such as exported serine protease gene *htrA* (A9497_06005 and AKL23_09565) and foldase protease gene *prsA* (A9497_08370 and AKL23_02295), which is responsible for degradation of misfolded exported proteins for growth at heat shock and protease maturation, respectively [23,24]. Moreover, genes encoding extracellular carboxypeptidase were identified in the two genomes, but only one gene is functional, because others were pseudogenes.

**Figure 4:**
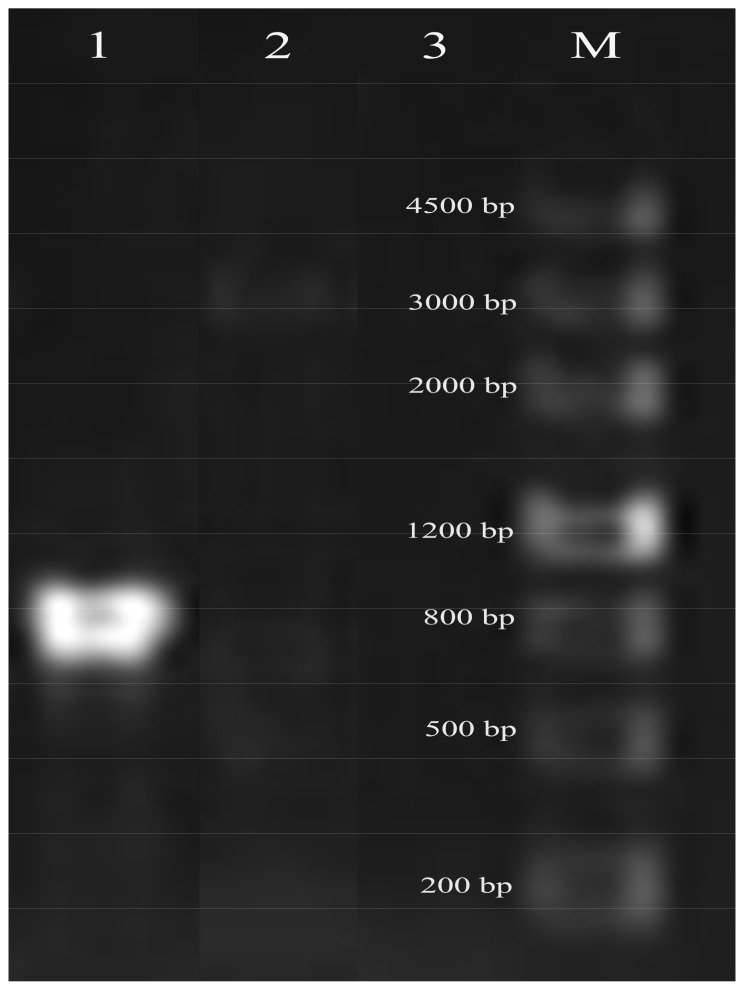
Electrophoresis of *prtS* gene in *S. thermophilus.* Lane 1: *S. thermophilus* KLDS SM; Lane 2: *S. thermophilus* KDLS3.1012; Lane 3: negative control without the template DNA and Lane M: marker.

For extracellular protease, *S. thermophilus* KLDS SM possess an intact CEP PrtS (A9497_00420), which is a key component for the cleavage of casein to oligopeptides and belongs to the subtilisin-like serine protease family [10]. Nevertheless, the *S. thermophilus* KLDS3.1012 genome does not carry this gene. The in silico analyses for the *prtS* gene were confirmed by PCR (Figure 4).

Oligopeptide and free amino acid transporters are important for the uptake of peptides and amino acids into cells. Genes encoding complete OppABCDF peptide transport system were present in the genomes of *S. thermophilus* KLDS SM and *S. thermophilus* KLDS3.1012. These two strains own the same genes encoding transporters for amino acid, branched-chain amino acid, polar amino acid, methionine, glutamine, threonine and serine/threonine as described in Table 2.

Once the peptides and amino acids are transported into *S. thermophilus* cells, and peptides will be further metabolized by various cytoplasmic peptidases, which hydrolyze them to free amino acids for cellular metabolism or for direct utilization [7,25]. Notably, these two strains possess a number of genes encoding cytoplasmic peptidases (Table 3), which are involved in carboxypeptidase, dipeptidases, endopeptidases, peptidase M16, peptidase M20 and aminopeptidases. Genes encoding intracellular protease including one gene encoding rhomboid family intramembrane serine protease and six genes encoding CAAX amino terminal protease were found in the genomes of these two strains. Additionally, different kinds of metalloprotease encoding genes were also characterized.

Overall, the prtS gene is the main difference in the proteolytic system between the two genomes; this could be the probable reason for phenotypic differences between the two strains behind the presence of prtS in the genome of S. thermophilus KLDS SM compared to prtS-deficient strain S. thermophilus KLDS3.1012. Phenotypic heterogeneity of S. thermophilus strains occurred via long-time adaptation to environment [26].

More recently, PrtS+ strains of *S. thermophilus* were observed to display two exponential growth phases in milk, and protease PrtS synthesis was initiated after the first exponential phase, leading to the accumulation of free amino acids and peptides in milk during the second exponential phase [27]. The presence of CEP PrtS has been highlighted to be directly linked to the high proteolytic activity, to the fast growth of *S. thermophilus*, and to the fast acidification of milk [14]. Our results from comparative genomics and phenotypic tests confirmed this view, again.

### 2.4. Special prtS Gene Analysis

Based on the importance of CEP PrtS, the special and key gene *prtS* (A9497_00420) in the genome of *S. thermophilus* KLDS SM was further analyzed in silico. Although CEP PrtS has been shown to endow a few monoculture strains with fast acidifying and rapid growth ability in milk [13,14], it is reported to be present only in a minority in this species studied to date [28]. As studied by the French National Institute for Agricultural Research (INRA), only 21 strains among the 135 strains of historical collection displayed a high level of protease activity [12]. In all 40 sequenced *S. thermophilus* strains, only 10 strains including KLDS SM, ASCC 1275, JIM 8232, LMD-9, MN-ZLW-002, MN-BM-A02, ND07, DGCC7110, ST3, C106, St-10 and TH 1435 harbor *prtS* gene by blastn. This ratio is higher than that ratio of INRA collection, which may result from the sequenced strains associated with attractive technological properties, such as optimal development in milk containing high protein content and rapid milk acidification.

**Figure 5:**
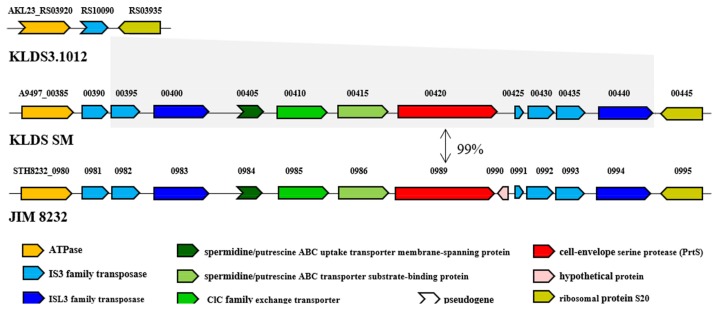
Chromosomal region targeted by the *prtS* gene in the genome of *S. thermophilus* KLDS3.1012, *S. thermophilus* KLDS SM and *S. thermophilus* JIM 8232.

The genomic islands of *S. thermophilus* KLDS SM were found by IslandViewer4. There are 15 genomic islands in the genome of *S. thermophilus* KLDS SM (Supplementary Materials Table S1), while the *prtS* gene (A9497_00420) is not located in any genomic islands. Comparative genomic analysis of *S. thermophilus* KLDS SM and *S. thermophilus* KLDS3.1012 illustrates that *S. thermophilus* KLDS SM owns a 15 kb specific region between the gene encoding ATPase and the gene encoding ribosomal protein S20 (Figure 5). It consists of the *prtS* gene and three genes *potC* (truncated), *potD* and *eriC* encoding a spermidine/putrescine ABC uptake transporter membrane-spanning protein, a spermidine/putrescine ABC transporter substrate-binding protein, and a ClC family exchange transporter, respectively. These genes are surrounded by IS elements of the IS3 family and ISL3 family, which bring about the emergence of horizontal gene transfer (HGT). Although one IS3 transposase was presented in the genome of *S. thermophilus* KLDS3.1012, it is a frameshifted pseudogene without the function of transposon.

### 2.5. Co-Culture of S. thermophilus and Lb. bulgaricus

Since *S. thermophilus* is generally found in mixed cultures, growth of PrtS-deficient strains relies on the use of oligopeptides released by another LAB such as *Lb*. *bulgaricus*, *Lactobacillus helveticus* or *Lactococcus lactis* [14]. In order to investigate the effect of PrtS on the symbiosis between yoghurt starter species, two strains of *Lb. bulgaricus* were used.

**Figure 6:**
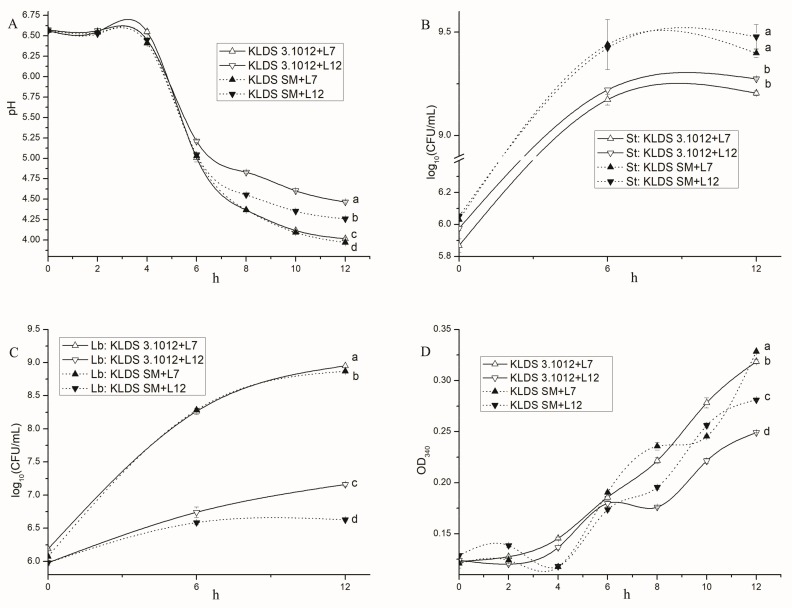
Comparison of technological properties of the four mixed cultures during milk fermentation: (**A**) pH curves of milk fermented by the four combinations; (**B**) Live counts of *S. thermophilus* in mixed culture during fermentation; (**C**) Live counts of *Lb. bulgaricus* in mixed culture; and (**D**) Proteolysis (OD340 nm) of milk fermented by the four combinations.

The *prtS*+ strain KLDS SM and *prtS*− strain KLDS3.1012 were combined respectively with two strains of *Lb. bulgaricus* (L7 and L12) which displayed different fermentation properties. The acidification (Figure 6A), survival of *S. thermophilus* (Figure 6B) and *Lb. bulgaricus* (Figure 6C), and proteolysis (Figure 6D) of yoghurt fermented by mixed cultures during 0–12 h were determined. The difference in acidification, growth, and proteolysis between our data and previous reports may be explained by the diversities of strains, milk composition, milk initial pH, bacterial inoculation rate, and cultivation time [13,15].

#### 2.5.1. Acid Production

The four combinations generally produced acid rapidly from ~4 h to 8 h. The two strongest acid-producing combinations were two combinations of L7 (KLDS SM + L7 and KLDS3.1012 + L7), followed by KLDS SM + L12. The proteolytic activity of KLDS SM increased the acid production and proteolysis of mixed cultures, especially in associations containing L12, which showed weak proteolytic activity. The pH divergence between KLDS SM + L7 and KLDS3.1012 + L7 during 10–12 h probably resulted from the caseins hydrolysis by KLDS SM. 

Three acidification phases of mixed culture in milk were observed in previous reports, which were corresponding to growth phases of *S. thermophilus* [15]. PrtB was largely related to the growth of *S. thermophilus*, but PrtS may play a part in its own growth when PrtB was absent or weak [15]. PrtS may be synthesized when free peptides and amino acids were exhausted in mixed culture [15,27].

#### 2.5.2. Bacterial Growth

*S. thermophilus* strains from the four associations shared similar growth curves, while *Lb. bulgaricus* strains were absolutely different. The counts of KLDS SM from its two combinations during stationary phase were significantly higher than KLDS3.1012 from the other combinations (*p* < 0.05). All counts of *Lb. bulgaricus* were below 10^9^ CFU·mL^−1^ during the 12 h fermentation in milk. L7 from its two associations displayed a significant increase in live counts, while L12 displayed slow growth rates. The counts of *Lb. bulgaricus* were found to be lower when combined with KLDS SM than with KLDS3.1012 during 6–12 h, suggesting that growth of *Lb. bulgaricus* may be influenced by the growth or proteolysis of *S. thermophilus*.

#### 2.5.3. Proteolysis

The two combinations containing L7 displayed higher proteolytic activities than the other combinations during 6–12 h, suggesting the proteolysis of mixed cultures is mainly depended by *Lb. bulgaricus*.

*S. thermophilus* with or without PrtS can efficiently consume the peptides by PrtB, and keep the dominant population during fermentation. This may because that *S. thermophilus* possesses higher levels of peptidases than *Lb. bulgaricus* [15,29]. And *Lb. bulgaricus* may require more complex casein degradation patterns than *S. thermophilus*. It was reported that cell count of *S. thermophilus* increased in reconstituted skim milk (RSM) when supplemented with milk peptide fractions, whereas no significant change (*p* > 0.05) in viable cells of *Lb. bulgaricus* was observed [30]. An earlier study pointed out that whey peptides and free amino acids promoted *Lb. bulgaricus* 2038 growth better than milk proteins [31], suggesting more factors that may influence growth of *Lb. bulgaricus*.

A pause of KLDS SM + L7 in the proteolysis curve during 8–10 h and then a rapid increase during 10–12 h were observed, probably because the consumption of free nitrogen source by KLDS SM used for expression of proteases PrtS.

#### 2.5.4. Effects of PrtS and PrtB on the Proto-Cooperation

Yoghurt is traditionally prepared through the proto-cooperation of *S. thermophilus* without PrtS and *Lb. bulgaricus* with strong proteolytic activities [13]. This symbiosis was known to benefit each other in growth, as well as to improve the acid production and flavor formation during yoghurt fermentation [15]. Recently, there has been an increased interest in PrtS+ strains of *S. thermophilus* for the application in high protein milk, rapid milk acidification, and acceleration of cheese ripening [14]. However, the stimulatory effect of PrtS was limited to *S. thermophilus* in monoculture [13,15]. And the benefit of bacterial association mainly depends on the employed *Lb. bulgaricus*, for PrtB is more efficient than PrtS in the supply of peptides to *S. thermophilus*, and different substrate specificity between PrtS and PrtB was affirmed [15]. However, relationship between the two species depends on the strains used, milk type, heating method, and fermentation temperature [32]. And proteolysis by *Lb. bulgaricus* was insufficient to meet the demands for sulfur and branched-chain amino acids by both strains [29].

### 2.6. Cold Storage of Yoghurts

The survival and proteolytic activities of *S. thermophilus* and *Lb. bulgaricus* contributed to the post-acidification of yoghurt throughout the shelf life [33]. *Lb. bulgaricus* was considered responsible for post-acidification due to its strong proteolytic activity and growth at low pH [18]. Mutant strains of *Lb. bulgaricus* were used to control the post-acidification [1,18].

**Figure 7:**
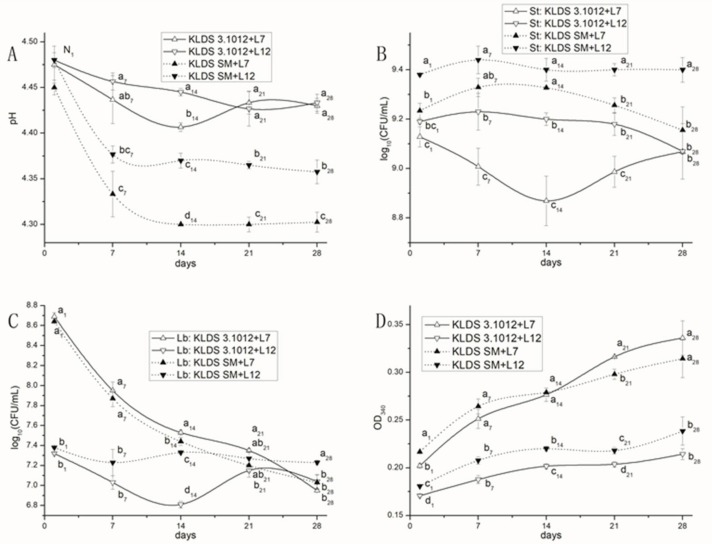
Comparison of industry properties of the four mixed cultures during cold storage: (**A**) pH curves of yoghurt produced by the four combinations during cold storage; (**B**) Survival of *S. thermophilus* in mixed culture; (**C**) Survival of *Lb. bulgaricus* in mixed culture; and (**D**) Proteolysis (OD340 nm) of yoghurt during cold storage. N1: no significant difference at 1st day.

Yoghurts (~pH 4.5) were prepared using different mixed cultures, and values of the pH (Figure 7A), live counts of *S. thermophilus* (Figure 7B) and *Lb. bulgaricus* (Figure 7C), and proteolysis (Figure 7D) throughout the 28 days storage at 4 °C were determined.

#### 2.6.1. Post-Acidification

The pH of yoghurts decreased sharply during the first 7 days of cold storage, but was relatively steady during 14–28 days. The pH values of the two combinations containing KLDS SM (KLDS SM + L7 and KLDS SM + L12) were significantly lower than the other two combinations (*p* < 0.05). Moreover, the pH values of KLDS SM + L7 were significantly lower than KLDS SM + L12 (*p* < 0.05).

Similar results were reported previously, showing a sharp decline in pH during 1–14 days of cold storage, but no change was found at 14–21 days [34]. The *prtS*+ strain KLDS SM greatly promoted the post-acidification of yoghurts during cold storage. PrtS is, however, indeed present in the natural and industrial environment, and mostly from cheese, indicating that the PrtS+ *S. thermophilus* can be used in the fermentation of milk containing high protein content and acceleration of cheese ripening [12,14,35].

#### 2.6.2. Survivals of Yoghurt Strains

The viabilities of *S. thermophilus* KLDS3.1012 and KLDS SM in the combinations were relatively constant throughout the 28 days of cold storage, while *Lb. bulgaricus* showed different survival patterns. The counts of L12 from its two associations were relatively steady. The survival of L7 from the other associations kept decrease during cold storage. 

The constant survivals of *S. thermophilus* and a significant decrease in counts of *Lb. bulgaricus* during cold storage were reported previously [36].

#### 2.6.3. Proteolysis

Proteolysis of the four yoghurt samples increased during the 28 days of cold storage, which was observed in a previous study [37]. The absorbance values of two associations containing L7 were significantly higher than the associations containing L12 (*p* < 0.05), indicating that proteolysis of yoghurt during cold storage was mainly influenced by the *Lb. bulgaricus* strains employed. The proteolytic activity persisting during the entire period of the shelf-life was considered an index of bacterial survival especially of *Lb. bulgaricus*, and free amino acids released from milk proteins are essential in the stimulation of streptococcus activity and in the formation of aroma compounds [38].

## 3. Materials and Methods

### 3.1. Strains and Culture Conditions

Two *S. thermophilus* strains (KLDS3.1012 and KLDS SM) and two *Lb. bulgaricus* strains (L7 and L12) were used in this study. All strains were available at the Key Laboratory of Dairy Science (KLDS), Northeast Agricultural University (NEAU), Harbin, China. KLDS3.1012 and KLDS SM were isolated from the fermented milk in Xinjiang and Inner Mongolia of China, respectively. L7 and L12 were isolated from the fermented milk in Qinghai and Xinjiang of China, respectively. Strains of *S. thermophilus* or *Lb. bulgaricus* were maintained in M17 or MRS broth with 10% glycerol and stored at −20 °C. Before use, *S. thermophilus* was subcultured three times (24 h per time) in M17 broth (Oxoid Ltd., Hampshire, UK) at 37 °C, and *Lb. bulgaricus* was subcultured three times (24 h per time) in de Man, Rogosa, and Sharpe (MRS) broth (Hopebio Technology Co., Ltd., Qingdao, China) at 42 °C [11,36]. The 10% (*w*/*v*) reconstituted skimmed milk was prepared using the NZMP skimmed milk powder (Fonterra Co-operative Group Limited, Oakland, New Zealand), and adjusted to pH 6.6 using HCl, and sterilized at 115 °C for 15 min [13,39].

### 3.2. Phenotype Terms of Single Strains

The pH, viability, and proteolysis during growth of single strain of *S. thermophilus* and *Lb. bulgaricus* were determined. For the pH and viability, strains were inoculated into milk with a rate of 1% (*v*/*v*) and incubated at 42 °C for 12 h. The pH and viability of each strain was monitored every 3 h. For proteolysis investigation, the revitalized strains were harvested by centrifugation at 10,000× *g* for 10 min, and washed twice and re-suspended to the original volume with phosphate-buffered saline (PBS) (pH 7.2), and then inoculated into milk, the rate of inoculation was 1% (*v*/*v*) and strains were incubated at 42 °C for 12 h. The proteolysis was determined at 0, 6, and 12 h.

#### 3.2.1. Enumeration of Viable Strains and pH Determination

Samples (1 mL) were mixed with 9 mL of sterile PBS, and subsequently, serial dilutions were used for enumeration of strains. The counts of *S. thermophilus* and *Lb. bulgaricus* in pure culture were enumerated using M17 and MRS agars (pH 6.2), while the individual populations of *S. thermophilus* and *Lb. bulgaricus* in mixed culture were enumerated using M17 agar and MRS agar (pH 5.2), respectively [36]. The M17 used for enumeration of *S. thermophilus* was incubated at 37 °C for 48 h, while the MRS (pH 6.2) and MRS (pH 5.2) used for enumeration of *Lb. bulgaricus* was incubated at 42 °C for 72 h. Colony forming units (CFU) were enumerated in plates containing 30–300 colonies, and cell concentration was expressed as log CFU·mL^−1^ of dairy samples [36,40]. The pH of dairy samples was determined directly using a METTLER TOLEDO EL20-K pH metre.

#### 3.2.2. Proteolysis

The proteolysis of samples was carried out as reported previously with some modifications [11]. Samples (5 mL each) were mixed with 1 mL H_2_O and 10 mL 0.75 M trichloroacetic acid (TCA), and incubated at room temperature (~22 °C) for 10 min followed by centrifugation at 13,000× *g* for 15 min. The supernatant obtained was stored at −20 °C before use. The proteolytic activity was measured by the determination of free amino groups, using the *o*-phthaldialdehyde (OPA) method, and the reaction reagents were prepared at the same day [41]. OPA reagent (1 mL) was added into 50 µL supernatant and incubated at room temperature (~22 °C) for 2 min, and absorbance was measured at 340 nm.

### 3.3. Genome Sequencing of S. thermophilus and Analysis in Silico

The genomic DNAs of the various strains used in this study were extracted using the TIANGEN TIANamp bacteria DNA kit (DP302) following the manufacturer’s instruction. The details of *S. thermophilus* KLDS SM genome sequencing, assembly and annotation were described by our previous report [42]. The genome of *S. thermophilus* KLDS3.1012 was sequenced by using the combining strategy of Illumina Hiseq (2 × 100 bp paired-end libraries, Illumina, San Diego, CA, USA) and Illumina Miseq (2 × 250 bp paired-end libraries) platform. Subsequently, 933 M Hiseq and 256 M Miseq clean data were produced through quality control and data filter. All the clean data were de novo assembled by SOAPdenovo2.0 [43]. Genome annotation was executed by NCBI Prokaryotic Genome Annotation Pipeline (Available online: http://www.ncbi.nlm.nih.gov/books/NBK174280). WebMGA (Available online: http://weizhong-lab.ucsd.edu/metagenomic-analysis/server/cog/) was used for COG annotation [44]. Circular genome map was generated by CGView Server [45]. ISfinder and IslandView4 were used for the identification of transposase and genomic island in the genome, respectively [46,47]. The functional regions were analyzed by Pfam 31.0 with an *E*-value of 1.0 [48].

### 3.4. Detection of prtS in S. thermophilus

The *prtS* in two strains of *S. thermophilus* was confirmed by PCR as previously reported [14]. PCR was operated using TIANGEN 2× Taq PCR MasterMix containing Taq DNA polymerase. Primers used in the present study were listed in Table S2. The temperature profile of the *prtS* gene was as follows: a pre-denaturation step was carried out at 94 °C for 5 min, and followed by 30 cycles of 94 °C for 1 min, 50 °C for the 30 s, 72 °C for 1 min, and finally 72 °C for 7 min.

### 3.5. Co-Culture

#### 3.5.1. Preparation of Starter Cultures

Single strains of *S. thermophilus* and *Lb. bulgaricus* were obtained firstly by the concentration of the culture volume. Strains of *S. thermophilus* and *Lb. bulgaricus* were cultivated in M17 and MRS broths (pH 6.5) for 18 h, respectively [49], and harvested by centrifugation at 7000× *g* for 10 min using the 500 mL centrifuge tubes, washed twice with phosphate-buffered saline (PBS) (pH 7.2), then re-suspended in 5 mL glycerol solution (1 M) and stored in −20 °C before use [50,51]. The viability of the starter cultures was obtained by the spread plate technique using M17 and MRS agar respectively prior to use.

#### 3.5.2. Fermentation and Cold Storage of Yoghurt

Four different types of mixed cultures were investigated in this study: (i) KLDS3.1012-L7; (ii) KLDS3.1012-L12; (iii) KLDS SM-L7; (iv) KLDS SM-L12. The combinations were inoculated into skimmed milk at a 1:1 ratio (1 × 10^6^:1 × 10^6^ CFU·mL^−1^), and cultivated at 42 °C for 12 h in the fermentation phase. The changes in pH, viability of bacterial strains and proteolysis were monitored as described by [13]. For the cold storage phase, the four combinations were first fermented until pH 4.5, and then transferred to 4 °C for 28 days. Afterwards, samples were analyzed for changes in pH, viability, and proteolysis at 1, 7, 14, 21, and 28 days of storage at 4 °C [52].

### 3.6. Data Treatment

All phenotype determinations of single and mixed cultures were carried out in triplicate, and data was analyzed by IBM SPSS Statistics software (Version 16.0, SPSS Inc., Chicago, IL, USA) with one-way ANOVA with a *p*-value is 0.05.

## 4. Conclusions

The effect of protease PrtS on yoghurt starter development was studied. A comparative genomics approach suggested that the *prtS* gene is the main difference in proteolytic systems between the two genomes of *S. thermophilus*. The PrtS showed positive effects on the acid production of mixed cultures during milk fermentation. But the acceleration depended on the proteolytic activity of *Lb. bulgaricus* strain employed. The PrtS also increased the post-acidification of yoghurts during cold storage, in accordance with its isolation from cheese and the application in cheese flavor formation. Results of the present study showed that The PrtS+ strains of *S. thermophilus* can be used to ferment milk with high content protein, to accelerate the ripening of cheese, or to develop the mild yoghurt combined with weak post acidification. Transcriptomics or proteomics will be used in the future to check the kinds of genes participating in casein hydrolysis and free nitrogen utilization, and the expression abundance of proteases and peptidases.

## Figures and Tables

**Table 1:** Extracellular protease and peptidase in strains KLDS SM and KLDS3.1012.

Encoded Proteins	Genes of Strains	
	KLDS SM	KLDS3.1012
Exported serine protease HrtA	A9497_06005	AKL23_RS09565
Protease maturation protein foldase PrsA	A9497_08370	AKL23_RS02295
Cell envelope serine proteinase PrtS	A9497_00420	−
d-alanyl-d-alanine carboxypeptidase	A9497_06565, ψA9497_06560, ψA9497_01445	AKL23_RS00470, ψAKL23_RS09800

ψ presents pseudogene; − presents no corresponding genes.

**Table 2:** Peptide and amino acid transport systems in strains KLDS SM and KLDS3.1012.

Specificity	Genes of Strains		Product
	KLDS SM	KLDS3.1012	
Oligopeptide	A9497_03170 A9497_03160	AKL23_RS06760 AKL23_RS06745	OppA; Substrate-binding protein
	A9497_03155	AKL23_RS06740	OppB; Permease protein
	A9497_03150	AKL23_RS06735	OppC; Permease protein
	A9497_03145	AKL23_RS06730	OppD; ATP-binding protein
	A9497_03140	AKL23_RS06725	OppF; ATP-binding protein
Amino acid	A9497_00725 A9497_04145	AKL23_RS04220 AKL23_RS07755	ATP-binding cassette (ABC) transporter ATP-binding protein
	A9497_00720 A9497_04150 A9497_03405 A9497_03795	AKL23_RS04215 AKL23_RS07760 AKL23_RS07020 AKL23_RS07405	ABC transporter permease
	A9497_04155 A9497_03360 A9497_03370 A9497_07620	AKL23_RS07765 AKL23_RS06975 AKL23_RS06985 AKL23_RS06990	ABC transporter substrate-binding protein
	A9497_03375 A9497_03785 A9497_07595	AKL23_RS07395 AKL23_RS01465 AKL23_RS01490	
Amino acid	A9497_08630	AKL23_RS02545	Transporter
	A9497_02760 A9497_03620 A9497_09055	AKL23_RS02000 AKL23_RS02915 AKL23_06320	Permease
Branched-chain amino acid	A9497_00490 A9497_01930 A9497_07910 A9497_07915	AKL23_RS01775 AKL23_RS01780 AKL23_RS05525 AKL23_RS08285	ABC transporter permease
	A9497_04685 A9497_04690	AKL23_RS08280 AKL23_RS08285	Permease
	A9497_07905	AKL23_RS01770	ABC transporter substrate-binding protein
Glutamine	A9497_01790 A9497_06995 A9497_00730	AKL23_RS04225 AKL23_RS00860 AKL23_RS05335	ABC transporter substrate-binding protein
	A9497_01795 A9497_01800 A9497_09175	AKL23_RS05340 AKL23_RS05345 AKL23_RS03035	ABC transporter permease
	A9497_03790 A9497_09180	AKL23_RS03040 AKL23_RS07400	ABC transporter ATP-binding protein
Methionine	A9497_07635	AKL23_RS01505	ABC transporter ATP-binding protein
	A9497_07640	AKL23_RS01510	ABC transporter permease
Polar amino acid	A9497_03800	AKL23_RS07410	ABC transporter permease
Serine/threonine	A9497_07645	AKL23_RS01515	Transporter SstT
Threonine	A9497_02865	AKL23_RS06450	Transporter RhtB

**Table 3:** Intracellular protease and peptidase in strains KLDS SM and KLDS3.1012.

Encoded Proteins	Genes of Strains	
	KLDS SM	KLDS3.1012
Rhomboid family intramembrane serine protease	A9497_05030	AKL23_RS08625
Membrane-bound protease2C CAAX family	A9497_04220 A9497_05120 A9497_05125 A9497_05130 A9497_05135 ψA9497_09495	AKL23_RS07830 AKL23_RS08710 AKL23_RS08715 AKL23_RS08720 AKL23_RS08725 ψAKL23_RS10450
Serine protease	A9497_00565	AKL23_RS04060
C3-degrading protease	A9497_04215	AKL23_RS07825
CPBP family intramembrane metalloprotease	A9497_09585	AKL23_RS03380
Metalloprotease	A9497_07170	AKL23_RS01030
Putative Zn-dependent protease	A9497_01910	AKL23_RS05505
ATP-dependent Zn protease	A9497_02810	AKL23_RS06395
Zinc protease	A9497_00005	AKL23_RS03520
Aminopeptidase T	A9497_06485	AKL23_RS00400
Aminopeptidase N	A9497_01225	AKL23_RS04750
Aminopeptidase C	A9497_07295	AKL23_RS01165
Xaa-Pro aminopeptidase	A9497_09300 A9497_04560	AKL23_RS08155 AKL23_RS03160
Tripeptide aminopeptidase	A9497_01685	AKL23_RS05230
Glutamyl aminopeptidase	A9497_05110	AKL23_RS08700
Methionine aminopeptidase	A9497_03680	AKL23_RS07290
d-alanyl-d-alanine carboxypeptidase	A9497_06720	AKL23_RS00620
Dipeptidase	A9497_01640 ψA9497_00590	AKL23_RS04085 ψAKL23_RS10110
Xaa-Pro dipeptidyl-peptidase	A9497_04230	AKL23_RS07840
Metalloendopeptidases	A9497_03000	AKL23_RS06585
Endopeptidase	A9497_05285	AKL23_RS08875
Oligoendopeptidase F	A9497_02650, A9497_08360	AKL23_RS02285
*O*-sialoglycoprotein endopeptidase	A9497_04050 A9497_07625	AKL23_RS07660 AKL23_RS01495
Peptidase M16	A9497_05935 A9497_05940	AKL23_RS09495 AKL23_RS09500
Peptidase M20	A9497_08905 ψA9497_07630	AKL23_RS02765 ψAKL23_RS01500

ψ presents pseudogene.

## Supplementary Materials

Supplementary materials can be found at http://www.mdpi.com/1422-0067/19/4/1068/s1.
