# Supplementary material for: Technological and Genomic Analysis of Roles of the Cell-Envelope Protease PrtS in Yoghurt Starter Development
Source: Int J Mol Sci. 2018 Apr 3;19(4):1068. doi: 10.3390/ijms19041068 (PMC5979370; doi:10.3390/ijms19041068)
Supplement: Supplementary file 1 [file ijms-19-01068-s001.pdf]

Table S1 The genomic islands of *S. thermophilus* KLDS SM

Start	End	Locus	Gene start	Gene end	Product
53781	61460	A9497_RS00270	53781	53969	hypothetical protein
53781	61460	A9497_RS00275	54248	54523	hypothetical protein
53781	61460	A9497_RS00280	54540	54725	hypothetical protein
53781	61460	A9497_RS00285	55023	56528	DNA primase
53781	61460	A9497_RS00290	56518	57378	hypothetical protein
53781	61460	A9497_RS00295	57393	57665	hypothetical protein
53781	61460	A9497_RS00300	57908	58144	hypothetical protein
53781	61460	A9497_RS00305	58158	58352	hypothetical protein
53781	61460	A9497_RS00310	58356	58649	hypothetical protein
53781	61460	A9497_RS00315	58888	59085	transcriptional regulator
53781	61460	A9497_RS00320	59248	59496	Cro/Cl family transcriptional regulator
53781	61460	A9497_RS00325	59843	61009	integrase
53781	61460	A9497_RS00330	61125	61460	hypothetical protein
115340	133684	A9497_RS00605	115340	116209	transposase
115340	133684	A9497_RS00610	116224	116910	transposase
115340	133684	A9497_RS00615	117105	118282	transposase
115340	133684	A9497_RS00620	118403	120493	copper-translocating P-type ATPase
115340	133684	A9497_RS00625	120684	121858	transposase
115340	133684	A9497_RS00630	122053	122809	type I restriction endonuclease
115340	133684	A9497_RS00635	123704	124615	cysteine synthase
115340	133684	A9497_RS00640	124637	125821	cystathionine beta-lyase
115340	133684	A9497_RS00645	125904	126131	serine acetyltransferase
115340	133684	A9497_RS00650	126872	128046	transposase
115340	133684	A9497_RS00655	128159	129046	sugar transporter
115340	133684	A9497_RS00660	129662	129856	hypothetical protein
115340	133684	A9497_RS00665	129925	130368	restriction endonuclease subunit M
115340	133684	A9497_RS00670	130678	131370	protease PrsW
115340	133684	A9497_RS00675	131527	133071	zinc ABC transporter substrate-binding protein AdcA
115340	133684	A9497_RS00680	133286	133684	hypothetical protein
122053	128046	A9497_RS00630	122053	122809	type I restriction endonuclease
122053	128046	A9497_RS00635	123704	124615	cysteine synthase
122053	128046	A9497_RS00640	124637	125821	cystathionine beta-lyase
122053	128046	A9497_RS00645	125904	126131	serine acetyltransferase
122053	128046	A9497_RS00650	126872	128046	transposase
169624	176090	A9497_RS00875	169624	170481	MutR family transcriptional regulator
169624	176090	A9497_RS00880	170541	171299	Fe-S oxidoreductase
169624	176090	A9497_RS00885	171334	172509	transposase
169624	176090	A9497_RS00890	173074	174171	radical SAM/SPASM domain-containing protein
169624	176090	A9497_RS00895	174168	174911	agmatinase
169624	176090	A9497_RS00900	174918	176090	MFS transporter

271805	280214	A9497_RS01390	271432	272373	subtype I-E CRISPR-associated endonuclease Cas1
271805	280214	A9497_RS01395	272377	273015	type I-E CRISPR-associated protein Cas6/Cse3/CasE
271805	280214	A9497_RS01400	273020	273745	type I-E CRISPR-associated protein Cas5/CasD
271805	280214	A9497_RS01405	273759	274826	type I-E CRISPR-associated protein Cas7/Cse4/CasC
271805	280214	A9497_RS01410	274816	275409	type I-E CRISPR-associated protein Cse2/CasB
271805	280214	A9497_RS01415	275419	277086	type I-E CRISPR-associated protein Cse1/CasA
271805	280214	A9497_RS01420	277073	279853	CRISPR-associated helicase/endonuclease Cas3
271805	280214	A9497_RS01425	280150	282093	hypothetical protein
516264	521871	A9497_RS02645	516264	517493	MFS transporter permease
516264	521871	A9497_RS02650	517471	519804	oligoendopeptidase F
516264	521871	A9497_RS02655	520178	521053	XRE family transcriptional regulator
516264	521871	A9497_RS02660	521182	521587	DNA mismatch repair protein MutT
516264	521871	A9497_RS02665	521587	521871	hypothetical protein
562306	569987	A9497_RS02885	562306	562980	peptide ABC transporter ATP-binding protein
562306	569987	A9497_RS02890	563723	563992	hypothetical protein
562306	569987	A9497_RS02895	564152	564451	hypothetical protein
562306	569987	A9497_RS02900	564551	565462	sodium transporter
562306	569987	A9497_RS02905	565459	565781	hypothetical protein
562306	569987	A9497_RS02910	565778	566353	SPASM domain-containing protein
562306	569987	A9497_RS02915	566474	567565	transporter
562306	569987	A9497_RS02920	567562	568882	KxxxW cyclic peptide radical SAM maturase
562306	569987	A9497_RS02925	569127	569987	MutR family transcriptional regulator
564552	569061	A9497_RS02900	564551	565462	sodium transporter
564552	569061	A9497_RS02905	565459	565781	hypothetical protein
564552	569061	A9497_RS02910	565778	566353	SPASM domain-containing protein
564552	569061	A9497_RS02915	566474	567565	transporter
564552	569061	A9497_RS02920	567562	568882	KxxxW cyclic peptide radical SAM maturase
837601	844715	A9497_RS04255	837601	838287	transposase
837601	844715	A9497_RS04260	838302	839171	transposase
837601	844715	A9497_RS04265	839317	839571	hypothetical protein
837601	844715	A9497_RS04270	839822	840223	hypothetical protein
837601	844715	A9497_RS04275	840817	841032	hypothetical protein
837601	844715	A9497_RS04280	841228	841458	bacteriocin
837601	844715	A9497_RS04285	841814	842014	hypothetical protein
837601	844715	A9497_RS04290	842033	842209	bacteriocin
837601	844715	A9497_RS04295	842465	843196	DNA-binding response regulator
837601	844715	A9497_RS04300	843196	844536	histidine kinase
837601	844715	A9497_RS04305	844554	844715	bacteriocin leader domain-containing protein
942826	951515	A9497_RS04755	942315	942850	transposase

942826	951515	A9497_RS04760	942826	943164	transposase
942826	951515	A9497_RS04765	943178	943636	transposase
942826	951515	A9497_RS04770	943748	944332	transposase
942826	951515	A9497_RS04775	944500	945003	transposase
942826	951515	A9497_RS04780	945038	946048	type I glyceraldehyde-3-phosphate dehydrogenase
942826	951515	A9497_RS04785	946235	948316	elongation factor G
942826	951515	A9497_RS04790	948537	949007	30S ribosomal protein S7
942826	951515	A9497_RS04795	949026	949439	30S ribosomal protein S12
942826	951515	A9497_RS04800	949656	950480	pur operon repressor
942826	951515	A9497_RS04805	950574	951515	3'-5' exoribonuclease YhaM
942826	951515	A9497_RS04810	951505	952779	DNA polymerase V
1050630	1054700	A9497_RS05370	1050005	1051600	type I restriction-modification system subunit M
1050630	1054700	A9497_RS05375	1051600	1054677	DEAD/DEAH box helicase
1050630	1054700	A9497_RS05380	1054688	1054981	hypothetical protein
1060633	1096101	A9497_RS05420	1060633	1061319	transposase
1060633	1096101	A9497_RS05425	1061517	1061903	50S ribosomal protein L17
1060633	1096101	A9497_RS05430	1061921	1062859	DNA-directed RNA polymerase subunit alpha
1060633	1096101	A9497_RS05435	1062908	1063291	30S ribosomal protein S11
1060633	1096101	A9497_RS05440	1063319	1063684	30S ribosomal protein S13
1060633	1096101	A9497_RS05445	1063844	1064062	translation initiation factor IF-1
1060633	1096101	A9497_RS05450	1064180	1064836	adenylate kinase
1060633	1096101	A9497_RS05455	1064968	1066263	preprotein translocase subunit SecY
1060633	1096101	A9497_RS05460	1066280	1066720	50S ribosomal protein L15
1060633	1096101	A9497_RS05465	1066848	1067030	50S ribosomal protein L30
1060633	1096101	A9497_RS05470	1067045	1067539	30S ribosomal protein S5
1060633	1096101	A9497_RS05475	1067558	1067914	50S ribosomal protein L18
1060633	1096101	A9497_RS05480	1068004	1068540	50S ribosomal protein L6
1060633	1096101	A9497_RS05485	1068667	1069065	30S ribosomal protein S8
1060633	1096101	A9497_RS05490	1069188	1069373	30S ribosomal protein S14 type Z
1060633	1096101	A9497_RS05495	1069391	1069933	50S ribosomal protein L5
1060633	1096101	A9497_RS05500	1069960	1070265	50S ribosomal protein L24
1060633	1096101	A9497_RS05505	1070346	1070714	50S ribosomal protein L14
1060633	1096101	A9497_RS05510	1070739	1070999	30S ribosomal protein S17
1060633	1096101	A9497_RS05515	1071027	1071233	50S ribosomal protein L29
1060633	1096101	A9497_RS05520	1071243	1071656	50S ribosomal protein L16
1060633	1096101	A9497_RS05525	1071660	1072313	30S ribosomal protein S3
1060633	1096101	A9497_RS05530	1072326	1072670	50S ribosomal protein L22
1060633	1096101	A9497_RS05535	1072686	1072964	30S ribosomal protein S19
1060633	1096101	A9497_RS05540	1073058	1073891	50S ribosomal protein L2
1060633	1096101	A9497_RS05545	1073909	1074205	50S ribosomal protein L23
1060633	1096101	A9497_RS05550	1074205	1074828	50S ribosomal protein L4
1060633	1096101	A9497_RS05555	1074853	1075479	50S ribosomal protein L3
1060633	1096101	A9497_RS05560	1075596	1075904	30S ribosomal protein S10
1060633	1096101	A9497_RS05565	1076148	1077149	Holliday junction DNA helicase RuvB

1060633	1096101	A9497_RS05570	1077232	1079055	acyltransferase
1060633	1096101	A9497_RS05575	1079052	1079462	membrane protein
1060633	1096101	A9497_RS05580	1079459	1079893	protein-tyrosine-phosphatase
1060633	1096101	A9497_RS05585	1080176	1081468	adenylosuccinate synthetase
1060633	1096101	A9497_RS05590	1081723	1082239	DNA topology modulation protein FlaR
1060633	1096101	A9497_RS05595	1082529	1083698	MFS transporter
1060633	1096101	A9497_RS05600	1083691	1084746	bacteriocin biosynthesis protein
1060633	1096101	A9497_RS05605	1085039	1085248	hypothetical protein
1060633	1096101	A9497_RS05610	1085276	1086441	transposase
1060633	1096101	A9497_RS05615	1086825	1087697	MutR family transcriptional regulator
1060633	1096101	A9497_RS05620	1087782	1089941	hypothetical protein
1060633	1096101	A9497_RS05625	1089946	1091514	ABC transporter ATP-binding protein
1060633	1096101	A9497_RS05630	1092429	1093127	MutR family transcriptional regulator
1060633	1096101	A9497_RS05635	1093572	1094567	radical SAM protein
1060633	1096101	A9497_RS05640	1094560	1096101	ABC transporter ATP-binding protein
1081723	1098603	A9497_RS05590	1081723	1082239	DNA topology modulation protein FlaR
1081723	1098603	A9497_RS05595	1082529	1083698	MFS transporter
1081723	1098603	A9497_RS05600	1083691	1084746	bacteriocin biosynthesis protein
1081723	1098603	A9497_RS05605	1085039	1085248	hypothetical protein
1081723	1098603	A9497_RS05610	1085276	1086441	transposase
1081723	1098603	A9497_RS05615	1086825	1087697	MutR family transcriptional regulator
1081723	1098603	A9497_RS05620	1087782	1089941	hypothetical protein
1081723	1098603	A9497_RS05625	1089946	1091514	ABC transporter ATP-binding protein
1081723	1098603	A9497_RS05630	1092429	1093127	MutR family transcriptional regulator
1081723	1098603	A9497_RS05635	1093572	1094567	radical SAM protein
1081723	1098603	A9497_RS05640	1094560	1096101	ABC transporter ATP-binding protein
1081723	1098603	A9497_RS05645	1096505	1096864	hypothetical protein
1081723	1098603	A9497_RS05650	1097198	1097380	hypothetical protein
1081723	1098603	A9497_RS05655	1097426	1098403	replication initiation protein
1081723	1098603	A9497_RS05660	1098415	1098603	hypothetical protein
1116063	1130559	A9497_RS05770	1116063	1116617	hypothetical protein
1116063	1130559	A9497_RS05775	1117243	1117557	diacylglycerol kinase
1116063	1130559	A9497_RS05780	1117867	1118504	hypothetical protein
1116063	1130559	A9497_RS05785	1118548	1118919	hypothetical protein
1116063	1130559	A9497_RS05790	1119509	1119748	membrane protein
1116063	1130559	A9497_RS05795	1119811	1120377	hypothetical protein
1116063	1130559	A9497_RS05800	1120390	1120560	membrane protein
1116063	1130559	A9497_RS05805	1120575	1121132	general stress protein
1116063	1130559	A9497_RS05810	1121205	1121408	CsbD family protein
1116063	1130559	A9497_RS05815	1122007	1122498	phosphohydrolase
1116063	1130559	A9497_RS05820	1122815	1123990	transposase
1116063	1130559	A9497_RS05825	1124160	1124487	PadR family transcriptional regulator
1116063	1130559	A9497_RS05830	1124693	1125031	hypothetical protein
1116063	1130559	A9497_RS05835	1125148	1125903	hypothetical protein

1116063	1130559	A9497_RS05840	1125940	1128309	membrane protein
1116063	1130559	A9497_RS05845	1128438	1128977	TetR family transcriptional regulator
1116063	1130559	A9497_RS05850	1129055	1129417	membrane protein
1116063	1130559	A9497_RS05855	1129948	1130559	30S ribosomal protein S4
1179539	1195480	A9497_RS06085	1179027	1179563	transposase
1179539	1195480	A9497_RS06090	1179539	1179733	transposase
1179539	1195480	A9497_RS06095	1179837	1180379	transposase
1179539	1195480	A9497_RS06205	1187230	1188060	rod shape-determining protein MreC
1179539	1195480	A9497_RS06210	1188062	1188586	rod shape-determining protein MreD
1179539	1195480	A9497_RS06215	1188671	1190038	peptidoglycan hydrolase
1179539	1195480	A9497_RS06220	1190252	1191217	ribose-phosphate pyrophosphokinase
1179539	1195480	A9497_RS06225	1191232	1191411	hypothetical protein
1179539	1195480	A9497_RS06230	1192324	1193499	aminotransferase A
1179539	1195480	A9497_RS06235	1193486	1194259	DNA repair protein RecO
1179539	1195480	A9497_RS06240	1194476	1195480	phosphate acyltransferase
1659186	1667221	A9497_RS08735	1659186	1659539	hypothetical protein
1659186	1667221	A9497_RS08740	1659779	1660024	ABC transporter substrate-binding protein
1659186	1667221	A9497_RS08745	1660086	1660339	ABC transporter substrate-binding protein
1659186	1667221	A9497_RS08750	1660608	1660847	macrolide ABC transporter
1659186	1667221	A9497_RS08755	1660844	1661059	hypothetical protein
1659186	1667221	A9497_RS08760	1661349	1661852	50S ribosomal protein L10
1659186	1667221	A9497_RS08765	1661927	1662295	50S ribosomal protein L7/L12
1659186	1667221	A9497_RS08770	1662466	1662681	hypothetical protein
1659186	1667221	A9497_RS08775	1662709	1663722	transposase
1659186	1667221	A9497_RS08780	1664572	1665228	peptide ABC transporter ATP-binding protein
1659186	1667221	A9497_RS08785	1665250	1665603	hypothetical protein
1659186	1667221	A9497_RS08790	1665651	1666520	transposase
1659186	1667221	A9497_RS08795	1666535	1667221	transposase
1791414	1796026	A9497_RS09425	1791414	1791961	GNAT family acetyltransferase
1791414	1796026	A9497_RS09430	1792141	1792707	hypothetical protein
1791414	1796026	A9497_RS09435	1792887	1793069	hypothetical protein
1791414	1796026	A9497_RS09440	1793551	1793778	hypothetical protein
1791414	1796026	A9497_RS09445	1793816	1795049	transposase
1791414	1796026	A9497_RS09450	1795344	1795604	transposase
1791414	1796026	A9497_RS09455	1795601	1796026	transposase
1845638	1851217	A9497_RS09705	1845638	1846765	hypothetical protein
1845638	1851217	A9497_RS09710	1846807	1847058	hypothetical protein
1845638	1851217	A9497_RS09715	1847355	1848092	hypothetical protein
1845638	1851217	A9497_RS09720	1848126	1849334	hypothetical protein
1845638	1851217	A9497_RS09725	1849349	1851217	NTPase

Table S2. Oligonucleotide primers used in detection of *prtS* of *S. thermophilus*

Gene	primer	Sequence
prtS	PrtSthF	ATACCTGCACCTTGTTGGCG
	PrtSthF	GGTTTCTGTTGTTATTGCAGC
